# 

**Published:** 1858-11

**Authors:** 


### AN INDEPENDENT JOURNAL,

DEVOTED TO

### Dental Progress, and Improvement in the Manufacture and Use of Instruments and Materials.

**EDITED BY JOHN T. TOLAND, 38 WEST FOURTH STREET,**

**CINCINNATI, O.**

### TERMS.

| | |
|---|---|
| One copy one year,........................................................ | 25 cents. |
| Five copies, one year to one address,.......................... | $1 00 |
| Thirty copies, one year to one address,........................ | 5 00 |
| One hundred copies, one year to one address,................ | 15 00 |

Those who will get up clubs, can thus realize a handsomo profit for themselves.

*All Subscriptions must be Cash in Advance*, as at this low price we can not afford to keep *book accounts*, nor incur the expense of sending out *collectors.* Every Dentist, Physician and Druggist should have it.

### TO ADVERTISERS,

The "REPORTER" offers superior advantages to any other Dental Journal, its circulation being much larger than any other, and from three to five times as great as some. The first No., 3,000, the present number 4,000 copies, and expect to increase with each issue.

The circulation extends to *every state* in the Union, but more especially *west* of the Allegheny mountains, where it goes to *every town* of any importance. It goes into the hands of the most INTELLIGENT and LIBERAL portion of the community, viz: *dentists*, *physicians* and *druggists*. Being a *book of reference*—it remains on the table in the offices of subscribers, where it is seen and frequently examined by the numerous visitors, who are waiting for operations or advice.

### TERMS OF ADVERTISING.

| | |
|---|---|
| One page for one year,......................................................... | $40 00 |
| Half a page, " ........................................................ | 25 00 |
| One fourth of a page, one year,........................................ | 15 00 |
| One eighth page, one year,................................................ | 10 00 |

*Special* advertisements by *contract.* Advertisements to be *paid in advance.*

Those who wish to avail themselves of the facilities of *extensive* advertising at *small expense* as above, will please forward their copy, accompanied by a *remittance* to *pay* for the amount of *space* they wish to occupy, according to the above terms, this will save the time and trouble of correspondence, and insure *prompt insertion.*

